# Serum Inflammatory Profile in Hereditary Transthyretin Amyloidosis: Mechanisms and Possible Therapeutic Implications

**DOI:** 10.3390/brainsci12121708

**Published:** 2022-12-12

**Authors:** Marco Luigetti, Angela Romano, Valeria Guglielmino, Maria Ausilia Sciarrone, Francesca Vitali, Carmine Carbone, Geny Piro, Andrea Sabino, Nicola De Stefano, Domenico Plantone, Guido Primiano

**Affiliations:** 1 Fondazione Policlinico Universitario A. Gemelli IRCCS, 00168 Rome, Italy; 2 Università Cattolica del Sacro Cuore, 00168 Rome, Italy; 3 Centre of Precision and Translation Medicine, Department of Medicine, Surgery and Neuroscience, University of Siena, 53100 Siena, Italy

**Keywords:** ATTRv, biomarker, inflammation, degeneration, therapy

## Abstract

Hereditary transthyretin (ATTRv) amyloidosis is a severe, progressive, and heterogeneous multisystemic condition due to mutations in the TTR gene. Although multiple aspects of its molecular pathophysiological mechanisms have been elucidated over the years, it is possible to hypothesize different pathogenetic pathways. Indeed, we extensively investigated the serum levels of several molecules involved in the immune response, in a cohort of ATTRv patients and healthy controls (HCs). Sixteen ATTRv patients and twenty-five HCs were included in the study. IFN-alpha levels were higher in ATTRv patients than in HCs, as well as IFN-gamma levels. By contrast, IL-7 levels were lower in ATTRv patients than in HCs. No significant difference between groups was found regarding IL-1Ra, IL-6, IL-2, IL-4, and IL-33 levels. Correlation analysis did not reveal any significant correlation between IFN-α, IFN-γ, IL-7, and demographic and clinical data. Larger and longitudinal studies using ultrasensitive methods to perform a full cytokine profiling are needed to better elucidate the role of inflammation in ATTRv pathogenesis and to test the reliability of these molecules as possible biomarkers in monitoring patients’ progression.

## 1. Introduction

Hereditary transthyretin (ATTRv) amyloidosis with polyneuropathy, also known as familial amyloid polyneuropathy (FAP), is a severe, progressive, and heterogeneous multisystemic condition due to mutations in the *TTR* gene. This autosomal-dominant neurogenetic disorder is characterized by an adult-onset with variable penetrance and a non-uniform phenotype, even in subjects carrying the same mutation [1,2]. Regarding molecular pathogenesis, ATTRv amyloidosis is a conformational disease caused by the aggregation of a specific protein, transthyretin (TTR), largely due to reduced folding stability and the consequent accumulation of insoluble amyloid fibrils through a dynamic process [3]. The extracellular deposition of amyloid in different organs, with a prevalent involvement of the somatic and autonomic peripheral nervous system (PNS), justifies the heterogeneity of the clinical manifestations of this hereditary disorder, in which extraneurological involvement is frequent for the cardiological, ocular, gastroenterological, and renal manifestations [4,5,6]. The prevalence of the disease is highly variable between endemic and non-endemic countries and the global prevalence ranges from 5526 to 38,468. However, the real numbers of ATTRv could even be higher, considering the missing diagnoses and the pre-symptomatic carriers regularly followed in each center [7].

Although multiple aspects of the molecular pathophysiological mechanisms associated with ATTRv amyloidosis have been elucidated over the years, particularly related to the TTR protein, it is possible to hypothesize different pathogenetic pathways. Despite the growing evidence in the literature regarding the central role of inflammatory mechanisms underlying neurodegenerative disorders, such as Parkinson’s disease and Alzheimer’s disease [8], only a few studies have been performed to date investigating this aspect in ATTRv amyloidosis with polyneuropathy [9,10,11]. In order to shed light on this specific aspect, we extensively investigated the serum levels of several molecules involved in the immune response, in a cohort of ATTRv patients to broaden the spectrum of the mechanisms involved in the development and progression of the disease.

## 2. Materials and Methods

### 2.1. Patient Population

Blood samples were collected from a case series (n = 16) of subjects with a confirmed pathogenic *TTR* variant and a diagnosis of ATTRv amyloidosis. Control samples were collected from 25 healthy volunteers without any evidence of neurologic, cardiac, renal, or autoimmune disease. The study was carried out according to the principles of the 1964 Declaration of Helsinki and its later amendments and approved by the Ethics Committee of Fondazione Policlinico Universitario A. Gemelli IRCCS, Roma, Italia (protocol ID 4409).

### 2.2. Cytokines Profiling

All serum specimens were analyzed for IL-1Ra, IL-2, IL-4, Il-6, IL-7, IL-33, IFN-α, and IFN-γ using Luminex XMAP multiplexing technology (Bioplex 200, Bio-Rad s.r.l.), according to the manufacturer’s instructions (Bio-Rad Laboratories). All assays were performed and Median fluorescence intensities were collected on a Luminex-200 instrument, using Bio-Plex Manager software version 6.2. Cytokines concentrations in samples were determined from the standard curve using a 5-point regression.

### 2.3. Clinical and Instrumental Evaluation

All patients underwent a complete neurological and neurophysiological evaluation by an expert neurologist, and several outcome measures were assessed, including familial amyloid polyneuropathy (FAP) stage, polyneuropathy disability (PND) score, the neuropathy impairment score (NIS), the quality of life-diabetic neuropathy (Norfolk QoL-DN) questionnaire and the compound autonomic dysfunction test (CADT). In addition, Sudoscan was performed on all enrolled patients according to previously described protocols [12]. Other outcome measures, such as interventricular septum (IVS) thickness and modified body mass index (mBMI), were also collected.

### 2.4. Statistical Analysis

Statistical analysis and graphs were generated with SPSS Statistics (IBM SPSS V.26, Chicago, IL, USA) and JMP (V.15, SAS Institute, Cary, NC, USA, 1989–2022). Data were summarized as frequencies (number/percentage) or median and interquartile range (IQR), as appropriate. Kolmogorov–Smirnov test was performed for the demonstration of normal distribution. When values were skewed, their levels were log transformed. Group differences for normally distributed data were assessed using analysis of variance. Quantitative data were compared with Fisher’s exact test. Analysis of covariance was performed by analyzing the log of analyte levels as dependent variables, groups (ATTRv patients and HCs) as fixed variables, and age and sex as covariates, to examine differences in terms of IL-1Ra, IL-2, IL-4, Il-6, IL-7, IL-33, and IFN-α and IFN-γ levels between the groups.

For those cytokines whose levels were significantly different in the ATTRv patients’ group as compared to healthy controls, a two-tailed Spearman’s rank-order correlation test was run to determine any linear relationship between them and demographic and clinical data in the group of ATTRv patients (disease duration, NIS, Norfolk QoL-DN questionnaire, CADT, lower and upper limbs’ Sudoscan, IVS thickness, mBMI).

A two-tailed *p* value of < 0.05 was considered statistically significant.

## 3. Results

A total of 41 subjects (16 ATTRv patients and 25 HCs) were included in the study. The median age of HCs was 50.0 years (IQR 46.5–59.5), and 48% were male. The median age of ATTRv patients was 69.5 years (IQR 62.25–75), and 81.25% were male. Patients with ATTRv were significantly older than HCs (*p* < 0.001), and the male-to-female ratio was different between the two groups (*p* = 0.026). Demographic and clinical data of patients are summarized in Table 1.

Cytokines levels in ATTRv patients and in HCs are shown in Table 2.

IFN-alpha levels were higher in ATTRv patients (median 1.03, IQR 0.63–1.28) than in HCs (median 0.0, IQR 0–0.12; *p* = 0.004), as well as IFN-gamma levels (ATTRv patients: median 4.24, IQR 3.09–4.95; HCs: median 0.0, IQR 0.0–1.07; *p* = 0.003). On the opposite, IL-7 levels were lower in ATTRv patients (median 4.35, IQR 3.70–5.73) than in HCs (median 6.55, IQR 5.84–8.45; *p* = 0.009). No significant difference between groups was found regarding IL-1Ra (ATTRv patients: median 518.77, IQR 416.98–981.42; HCs: median 587.67, IQR 440.41–842.70; *p* = 0.76), IL-6 (ATTRv patients: median 0.88, IQR 0.71–1.27; HCs: median 0.35, IQR 0.16–0.59; *p* = 0.09), IL-2 (ATTRv patients: median 0.30, IQR 0.02–0.83; HCs: median 0.91, IQR 0.58–1.16; *p* = 0.1), IL-4 (ATTRv patients: median 6.84, IQR 3.78–10.11; HCs: median 4.75, IQR 1.10–7.80; *p* = 0.41), and IL-33 (ATTRv patients: median 0.56, IQR 0.07–1.06; HCs: median 0.46, IQR 0.20–0.76; *p* = 0.99) levels.

Correlation analysis did not reveal any significant correlation between IFN-α, IFN-γ, IL-7, and demographic and clinical data (*p* > 0.05).

## 4. Discussion

In this prospective study, serum levels of IFN-alpha and IFN-gamma were found to increase, whereas Il-7 was found to decrease in ATTRv patients compared to HCs.

Historically, ATTRv amyloidosis has been viewed as a non-inflammatory disease, mainly due to the absence of any mononuclear cell infiltration in ex vivo tissues [9]. Nevertheless, few studies in the last two decades [10,11,12,13] shed light on the role played by the inflammatory response in ATTRv amyloidosis patients, although its precise contribution is still far from being understood in detail. Fibrillary TTR species bind to the receptor for advanced glycation end products (RAGE), activating the nuclear factor κB (NF-κB) pathway [14] which modulates various aspects of inflammation, and is critical for the macrophage inflammatory responses triggered by both the TRIF-dependent pathways that lead to the production of type I IFNs, IFN-alpha, and IFN-beta [15,16]. Moreover, NF-κB promotes Th1 cell differentiation, since Th1 cells are characterized by the secretion of IFN-γ [17]. In our patients, both IFN-alpha and IFN-gamma were increased, and this might be related to the ATTRv-induced immune response [10]. Both these molecules belong to the IFN family [18], IFN-alpha to the type I class and IFN-gamma being the only member of the type II class. Generally, IFN-alpha is mainly produced by leukocytes, mainly plasmacytoid dendritic cells [19]. IFN-gamma is predominantly produced by innate-like lymphocytes, such as activated natural killer cells in the acute phase, and by adaptive immune cells, such as activated T lymphocytes in the chronic phase of the immune response [20]. Interestingly, IFN-beta, another IFN belonging to the type I class, has also been found elevated in the sera of symptomatic ATTRv amyloidosis patients [10]. The roles of IFN-alpha [21,22] and IFN-gamma [23,24,25] have been extensively studied in the pathogenesis of several autoimmune diseases, including systemic lupus erythematosus, Sjögren syndrome, myositis, systemic sclerosis, and rheumatoid arthritis. Inversely, neuro-axonal degeneration has been demonstrated to induce a type I interferon response with dual protective and negative effects on its progression [26,27,28]. IFN-gamma has also a pivotal role in the progression of neurodegeneration, stimulating microglia proliferation, synapse elimination, and nitric oxide release that result in impaired synaptic transmission [20]. The available pathological studies that explored the presence of inflammatory cytokines in FAP nerve biopsies found higher levels of these molecules, especially localized to the endoneurial axons [10], but no significant white cell infiltration was demonstrated [10], making still uncertain the actual source of increased serum levels of IFN-alpha and IFN-gamma. We can either hypothesize that these cytokines primarily relate to the pathogenesis of the disease or that they are a secondary response to tissue injury.

On the contrary, serum levels of IL-7 were lower in our ATTRv amyloidosis patients. IL-7 has been widely known for its importance as a growth factor secreted by bone marrow stromal cells for B-cell progenitor survival and proliferation [29,30]. Indeed, IL-7 is also produced by the thymus and other epithelial cells, including keratinocytes and enterocytes, and significantly influence the development and homeostasis of several other immune cells, including T cells, natural killer cells, innate lymphoid cells, monocytes, macrophages and dendritic cells [31]. IL-7 has been demonstrated to boost T and B cell survival and activities, increasing antibody production [31]. In relation to ATTRv amyloidosis, no data is available on the B and T cell modifications and on the production of naturally occurring antibodies (Nabs) against ATTRv. Interestingly, in other diseases characterized by pathological protein deposition, including Alzheimer’s disease and Parkinson’s disease, Nabs targeting amyloid-beta (Nabs-Aβ) and alpha-synuclein (Nabs-α-Syn), respectively, are currently considered important in the pathogenesis of these diseases [32,33,34] and, even if their role is far from being fully clarified, Nabs are generally considered protective against neurodegeneration [34,35].

Compared to a previous study, we did not find any modification of IL-33 levels in our ATTRv patients [10]. In fact, the majority of patients recruited by Azavedo and colleagues were asymptomatic and mildly symptomatic patients and the increase did not remain significant if only moderately or severely symptomatic patients were considered. IL-33 functions as a nuclear alarmin, released from the nuclei of producing cells after the injury, to warn the immune cells of the damage [36,37]. Therefore, we can speculate that, in patients with an already established disease, the release of IL-33 becomes progressively less abundant than in the initial phase.

Finally, in our ATTRv patients, we did not find any modification of serum IL-6 levels, confirming the results of Azavedo and colleagues. In their study, these authors did not find any change in IL-6 levels in all the FAP disease stages compared to HCs [10]. However, a previous study [10] found increased serum concentration of IL-6 in FAP carriers and patients. When IL-6 levels are studied, the results should be analyzed considering the strict age-dependence of IL-6 levels [38] and the well-known circadian rhythm of IL-6 secretion in both young and older persons with two nadirs at about 8.00 and 21.00, and two zeniths at about 19.00 and 5.00 [38] that may significantly impact the results. Therefore, further studies need to be carefully planned, taking all these aspects into consideration, in order to avoid confusion by the time of day, to confirm whether peripheral blood IL-6 levels are elevated or not in ATTRv patients. This may be important, considering that IL-6 production is also mainly modulated by NF-κB and the multifaceted role that this cytokine plays in the immune response [38].

The results of our study also have an important possible therapeutic implication. Future longitudinal studies on patients starting from the asymptomatic phases may allow us to characterize the evolution of the inflammatory changes during the course of the disease. It would also be important to understand whether currently available drugs can influence the immune alterations herein described in ATTRv patients. From this perspective, should the results of future studies strengthen the conclusions of our work, the use of specific immunosuppressive therapies in these patients may be tested in clinical trials.

Our study is limited due to the small number of patients recruited and because the patient and control groups are not balanced with respect to age and sex. Patients with ATTRv were older than HCs, and the male-to-female ratio was different between the two groups.

## 5. Conclusions

In conclusion, the results of this pilot study suggest that ATTRv amyloidosis is characterized by a modification of serum levels of IFN-alpha, IFN-gamma, and IL-7. Larger and longitudinal studies using ultrasensitive methods to perform a full cytokine profiling are needed to better elucidate the role of inflammation in the pathogenesis of the disease and to test the reliability of these molecules as possible biomarkers in monitoring patients with ATTRv in clinical settings.

## Figures and Tables

**Table 1:** Detailed demographic and clinical data in our ATTRv cohort.

Subject and Sex	TTR Variant	Age at Onset	Age at Evaluation	FAP Stage	PND Score	Systemic Involvement	IVS (mm)	NIS	Norkfolk QoL-DN	CADT	Sudoscan LL (μS)	Sudoscan UL (μS)
M#1	F64L	72	75	1	2	GI	15	47,00	46	20	58	80
M#2	V32R	57	65	2	3b	H, Dys, K, GI	18	148,00	84	7	30	40
M#3	F64L	69	80	2	3a	H, Dys, GI	16	76,75	58	13	47	36
M#4	F64L	70	75	2	3a	H, Dys, GI	13	112,75	98	11	22	23
M#5	V30M	62	66	2	3a	//	10	65,00	47	17	26	56
M#6	V30M	58	66	2	3a	H, GI	13	98,00	52	18	31	45
M#7	V30M	64	69	1	2	H	15	69,50	73	17	31	71
M#8	V30M	64	75	1	1	H, GI	19	38,50	32	11	80	30
M#9	F64L	51	53	1	1	GI	9	28,50	18	19	76	73
F#10	F64L	58	60	1	1	//	10	23,00	13	15	75	79
M#11	F64L	63	70	2	3a	H, Dys, K, GI	22	77,75	100	15	45	67
F#12	F64L	75	75	1	1	//	12	2,00	56	13	59	71
M#13	V30M	54	54	1	1	H	15	12,00	2	20	76	89
F#14	F64L	61	69	1	2	Dys, GI	10	86,00	78	9	71	73
M#15	A109S	65	78	2	3b	Dys, GI	19	138,50	61	10	18	10
M#16	V30M	56	70	1	2	Dys, GI	17	92,00	46	11	19	24

**Legend:** TTR, transthyretin; FAP, Familial Amyloid Polyneuropathy; PND, Polyneuropathy Disability score; H, heart; Dys, dysautonomia; K, kidney; GI, gastro-intestinal; IVS, interventricular septum; NIS, Neuropathy Impairment Score; Norfolk QoL-DN, Norfolk Quality of Life-Diabetic Neuropathy questionnaire; CADT, Compound Autonomic Dysfunction Test; LL, lower limbs; UL, upper limbs.

**Table 2:** Cytokines levels (pg/mL) in ATTRv patients and healthy controls (HCs), expressed as median and interquartile range (IQR). Bold values denote statistical significance at the *p* < 0.05 level.

Examined Cytokine	ATTRv Patients (*N* = 16)	HCs (*N* = 25)	*p* value
IL-1Ra	518.77 (416.98–981.42)	587.67 (440.41–842.70)	0.760
IL-2	0.30 (0.02–0.83)	0.91 (0.58–1.16)	0.100
IL-4	6.84 (3.78–10.11)	4.75 (1.10–7.80)	0.410
IL-6	0.88 (0.71–1.27)	0.35 (0.16–0.59)	0.090
IL-7	4.35 (3.70–5.73)	6.55 (5.84–8.45)	**0.009**
IL-33	0.56 (0.07–1.06)	0.46 (0.20–0.76)	0.990
IFN-α	1.03 (0.63–1.28)	0.00 (0.00–0.12)	**0.004**
IFN-γ	4.24 (3.09–4.95)	0.00 (0.00–1.07)	**0.003**

## Data Availability

The data that support the findings of this study are available from the corresponding author, upon reasonable request.

## References

[1] Adams D., Koike H., Slama M., Coelho T. (2019). Hereditary transthyretin amyloidosis: A model of medical progress for a fatal disease. Nat. Rev. Neurol..

[2] Manganelli F., Fabrizi G.M., Luigetti M., Mandich P., Mazzeo A., Pareyson D. (2020). Hereditary Hereditary transthyretin amyloidosis overview. Neurol. Sci..

[3] Sekijima Y. (2015). Transthyretin (ATTR) amyloidosis: Clinical spectrum, molecular pathogenesis and disease-modifying treatments. J. Neurol. Neurosurg. Psychiatry.

[4] Ferraro P.M., D’Ambrosio V., Di Paolantonio A., Guglielmino V., Calabresi P., Sabatelli M., Luigetti M. (2021). Renal Involvement in Hereditary Transthyretin Amyloidosis: An Italian Single-Centre Experience. Brain Sci..

[5] Minnella A.M., Rissotto R., Maceroni M., Romano A., Fasciani R., Luigetti M., Sabatelli M., Rizzo S., Falsini B. (2021). Ocular Involvement in Hereditary Transthyretin Amyloidosis: A Case Series Describing Novel Potential Biomarkers. Genes.

[6] Luigetti M., Tortora A., Romano A., Di Paolantonio A., Guglielmino V., Bisogni G., Gasbarrini A., Calabresi P., Sabatelli M. (2020). Gastrointestinal Manifestations in Hereditary Transthyretin Amyloidosis: A Single-Centre Experience. J. Gastrointestin. Liver Dis..

[7] Luigetti M., Guglielmino V., Antonini G., Casali C., Ceccanti M., Chiappini M.G., De Giglio L., Di Lazzaro V., Di Muzio A., Goglia M. (2021). ATTRv in Lazio-Italy: A High-Prevalence Region in a Non-Endemic Country. Genes.

[8] Glass C.K., Saijo K., Winner B., Marchetto M.C., Gage F.H. (2010). Mechanisms underlying inflammation in neurodegeneration. Cell.

[9] Sousa M.M., Du Yan S., Fernandes R., Guimaraes A., Stern D., Saraiva M.J. (2001). Familial amyloid polyneuropathy: Receptor for advanced glycation end products-dependent triggering of neuronal inflammatory and apoptotic pathways. J. Neurosci..

[10] Azevedo E.P., Guimaraes-Costa A.B., Bandeira-Melo C., Chimelli L., Waddington-Cruz M., Saraiva E.M., Palhano F.L., Foguel D. (2019). Inflammatory profiling of patients with familial amyloid polyneuropathy. BMC Neurol..

[11] Gonçalves N.P., Vieira P., Saraiva M.J. (2014). Interleukin-1 signaling pathway as a therapeutic target in transthyretin amyloidosis. Amyloid.

[12] Luigetti M., Bisogni G., Romano A., Di Paolantonio A., Barbato F., Primicerio G., Rossini P.M., Servidei S., Sabatelli M. (2018). Sudoscan in the evaluation and follow-up of patients and carriers with TTR mutations: Experience from an Italian Centre. Amyloid.

[13] Suenaga G., Ikeda T., Masuda T., Motokawa H., Yamashita T., Takamatsu K., Misumi Y., Ueda M., Matsui H., Senju S. (2017). Inflammatory state exists in familial amyloid polyneuropathy that may be triggered by mutated transthyretin. Sci. Rep..

[14] Sousa M.M., Yan S.D., Stern D., Saraiva M.J. (2000). Interaction of the receptor for advanced glycation end products (RAGE) with transthyretin triggers nuclear transcription factor kB (NF-kB) activation. Lab. Investig..

[15] Wang N., Liang H., Zen K. (2014). Molecular mechanisms that influence the macrophage m1-m2 polarization balance. Front. Immunol..

[16] Liu T., Zhang L., Joo D., Sun S.C. (2017). NF-κB signaling in inflammation. Signal Transduct. Target Ther..

[17] Oh H., Ghosh S. (2013). NF-κB: Roles and regulation in different CD4^+^ T-cell subsets. Immunol. Rev..

[18] Walter M.R. (2020). The Role of Structure in the Biology of Interferon Signaling. Front. Immunol..

[19] Psarras A., Emery P., Vital E.M. (2017). Type I interferon-mediated autoimmune diseases: Pathogenesis, diagnosis and targeted therapy. Rheumatology.

[20] Kann O., Almouhanna F., Chausse B. (2022). Interferon γ: A master cytokine in microglia-mediated neural network dysfunction and neurodegeneration. Trends Neurosci..

[21] Niewold T.B., Clark D.N., Salloum R., Poole B.D. (2010). Interferon alpha in systemic lupus erythematosus. J. Biomed. Biotechnol..

[22] Muskardin T.L.W., Niewold T.B. (2018). Type I interferon in rheumatic diseases. Nat. Rev. Rheumatol..

[23] Theofilopoulos A.N., Koundouris S., Kono D.H., Lawson B.R. (2001). The role of IFN-gamma in systemic lupus erythematosus: A challenge to the Th1/Th2 paradigm in autoimmunity. Arthritis Res..

[24] Kato M. (2020). New insights into IFN-γ in rheumatoid arthritis: Role in the era of JAK inhibitors. Immunol. Med..

[25] Del Papa N., Minniti A., Lorini M., Carbonelli V., Maglione W., Pignataro F., Montano N., Caporali R., Vitali C. (2021). The Role of Interferons in the Pathogenesis of Sjögren’s Syndrome and Future Therapeutic Perspectives. Biomolecules.

[26] Khorooshi R., Owens T. (2010). Injury-induced type I IFN signaling regulates inflammatory responses in the central nervous system. J. Immunol..

[27] Hosmane S., Tegenge M.A., Rajbhandari L., Uapinyoying P., Ganesh Kumar N., Thakor N., Venkatesan A. (2012). Toll/interleukin-1 receptor domain-containing adapter inducing interferon-β mediates microglial phagocytosis of degenerating axons. J. Neurosci..

[28] Nazmi A., Field R.H., Griffin E.W., Haugh O., Hennessy E., Cox D., Reis R., Tortorelli L., Murray C.L., Lopez-Rodriguez A.B. (2019). Chronic neurodegeneration induces type I interferon synthesis via STING, shaping microglial phenotype and accelerating disease progression. Glia.

[29] Fistonich C., Zehentmeier S., Bednarski J.J., Miao R., Schjerven H., Sleckman B.P., Pereira J.P. (2018). Cell circuits between B cell progenitors and IL. Cell circuits between B cell progenitors and IL-7^+^ mesenchymal progenitor cells control B cell development. J. Exp. Med..

[30] Chen D., Tang T.X., Deng H., Yang X.P., Tang Z.H. (2021). Interleukin-7 Biology and Its Effects on Immune Cells: Mediator of Generation, Differentiation, Survival, and Homeostasis. Front. Immunol..

[31] Alpdogan O., van den Brink M.R. (2005). IL-7 and IL-15: Therapeutic cytokines for immunodeficiency. Trends Immunol..

[32] Bach J.P., Dodel R. (2012). Naturally occurring autoantibodies against β-Amyloid. Adv. Exp. Med. Biol..

[33] Liu Y.H., Wang J., Li Q.X., Fowler C.J., Zeng F., Deng J., Xu Z.Q., Zhou H.D., Doecke J.D., Villemagne V.L. (2021). Association of naturally occurring antibodies to β-amyloid with cognitive decline and cerebral amyloidosis in Alzheimer’s disease. Sci. Adv..

[34] Braczynski A.K., Sevenich M., Gering I., Kupreichyk T., Agerschou E.D., Kronimus Y., Habib P., Stoldt M., Willbold D., Schulz J.B. (2022). Alpha-Synuclein-Specific Naturally Occurring Antibodies Inhibit Aggregation In Vitro and In Vivo. Biomolecules.

[35] Qu B.X., Gong Y., Moore C., Fu M., German D.C., Chang L.Y., Rosenberg R., Diaz-Arrastia R. (2014). Beta-amyloid auto-antibodies are reduced in Alzheimer’s disease. J. Neuroimmunol..

[36] Cayrol C. (2021). IL-33, an Alarmin of the IL-1 Family Involved in Allergic and Non Allergic Inflammation: Focus on the Mechanisms of Regulation of Its Activity. Cells.

[37] Cayrol C., Girard J.P. (2022). Interleukin-33 (IL-33): A critical review of its biology and the mechanisms involved in its release as a potent extracellular cytokine. Cytokine.

[38] Maggio M., Guralnik J.M., Longo D.L., Ferrucci L. (2006). Interleukin-6 in aging and chronic disease: A magnificent pathway. J. Gerontol. A Biol. Sci. Med. Sci..

